# Retraction of: „lncRNA LENGA sponges miR-378 to promote myocardial fibrosis in atrial fibrillation”

**DOI:** 10.1515/med-2026-1553

**Published:** 2026-11-02

**Authors:** Liting Wu, Bingjing Gao, Mengyuan Shen, Lu Wei, Zhumeng Li, Wenfang Zhuang

**Affiliations:** Medical Laboratory, Shidong Hospital Affiliated to University of Shanghai for Science and Technology, Shanghai, China

Retraction of: Wu L, Gao B, Shen M, Wei L, Li Z, Zhuang W. lncRNA LENGA sponges miR-378 to promote myocardial fibrosis in atrial fibrillation. Open Medicine 2023; 18 (1): 20230831, DOI: 10.1515/med-2023-0831.

The retraction has been agreed by the Editor-in-Chief, Professor Vittorio Calabrese, and the publisher, Walter de Gruyter GmbH. The retraction is due to several scientific and editorial concerns related to the validity of the reported lncRNA annotation, the ceRNA mechanism, experimental controls, data transparency, and reporting standards.

